# Flow Behavior and Electrical Conductivity Characteristics of Carbonate Rocks Based on Pore-Scale Glass-Etched Experiments

**DOI:** 10.3390/mi17080897

**Published:** 2026-07-26

**Authors:** Qiang Lai, Junpeng Yao, Yuyu Wu, Bin Zhao, Xiuying Sui, Bing Xie, Chunlei Liu, Feng Wu

**Affiliations:** 1 Research Institute of Exploration and Development, Southwest Oil & Gas Field Company, PetroChina, Chengdu 610041, China; laiqiang@petrochina.com.cn (Q.L.); wuyuyu01@petrochina.com.cn (Y.W.); xxb-th@petrochina.com.cn (B.X.); 2 Geological Research Institute, China National Logging Corporation, Xi’an 710200, China; junpengyao@163.com (J.Y.); suixy2014@cnpc.com.cn (X.S.); liucl2014@cnpc.com.cn (C.L.); 3 School of Geoscience and Technology, Southwest Petroleum University, Chengdu 610500, China; 18298583363@163.com

**Keywords:** carbonate rock, glass-etched micromodel, gas–water displacement, water saturation, resistivity

## Abstract

Deep complex carbonate reservoirs contain different types of pore space, including vugs and fractures. Their gas–water distributions and flow characteristics are complex. The effect of pore structure and gas–water flow on resistivity in deep complex carbonate reservoirs remains unclear. This uncertainty creates challenges for natural gas exploration and development. A gas–water displacement and impedance synchronous measurement platform was established. Three types of glass-etched micromodels were fabricated from CT images of real carbonate cores. These models included a fracture–vuggy type with large aperture, a fracture–vuggy type with small aperture, and a vuggy type. After the micromodels were saturated with dyed formation water, gas–water displacement and water–gas displacement were conducted sequentially. Microscopic images of gas–water distribution, water saturation, resistivity, and resistivity index were obtained. The results showed that gas preferentially entered connected fractures and large pore throats, forming preferential channels. Residual water was mainly retained in vug corners, narrow throats, and weakly connected zones. During gas–water displacement, resistivity increased as water saturation decreased. The resistivity response showed a three-stage pattern of slow increase, rapid increase, and subsequent slow increase. During water–gas displacement, resistivity decreased as water saturation increased. The resistivity response showed a three-stage pattern of slow decrease, rapid decrease, and subsequent slow decrease. All three models exhibited non-Archie behavior. The stage-specific saturation exponent n ranged from 2.62 to 13.10, 4.36 to 7.24, and 5.37 to 9.84 for the fracture–vuggy type with large aperture, fracture–vuggy type with small aperture, and vuggy type, respectively. Their overall n values were 7.26, 6.36, and 5.39, showing that the fracture–vuggy type with large aperture had the most abrupt electrical response. This study clarified the relationship between gas–water flow characteristics and conductive response in carbonate rocks with different pore structures. The results provide pore-scale experimental evidence for water-invasion identification, remaining gas evaluation, and calibration of rock electrical parameters in complex carbonate reservoirs.

## 1. Introduction

Deep complex carbonate reservoirs exhibit strong heterogeneity and well-developed multi-scale pore space composed of pores, vugs, and fractures. The fluid occurrence state and migration pathways within these reservoirs are complex. As a result, gas–water two-phase flow differs substantially from that in conventional homogeneous porous media [1,2]. In fracture–vuggy carbonate reservoirs, pore structure controls preferential fluid-migration channels, residual-fluid distribution, and displacement efficiency. It also affects electrical response and the variation in rock electrical parameters. The relationship among pore structure, fluid distribution, and conductive response therefore needs to be clarified. Such clarification is important for understanding gas accumulation and development in carbonate gas reservoirs and for improving reservoir evaluation and dynamic interpretation [3,4].

Conventional core-displacement experiments are commonly used to characterize flow behavior at the macroscopic scale. However, rocks are opaque, and their internal pore structures are complex. Therefore, two-phase interface migration, local residual trapping, and preferential-channel formation are difficult to observe directly at the pore scale. High-resolution CT scanning and digital rock analysis provide effective tools for pore-structure reconstruction and flow-path analysis [5,6,7,8]. High-resolution CT and SEM imaging can further resolve mineral and pore features. For example, SEM imaging combined with numerical simulation has been used to connect microscale rock characteristics with engineering response [9]. Neutron scattering provides complementary information on pore-size distributions across multiple scales [10]. Pore-network modeling and pore-scale imaging can be used to characterize pore-throat structures and connectivity [11,12,13,14], as well as two-phase flow events [15,16,17,18]. Recent studies further show that fluid exposure and fluid–rock interactions can alter microscopic pore structure and cross-scale mechanical response in shale and coal [19,20]. Changes in wettability and cross-scale gas transport also demonstrate the coupling between microscale fluid behavior and pore-structure evolution [21,22]. Nevertheless, these methods are affected by image resolution, threshold segmentation, boundary conditions, and model assumptions. Flow results in complex fracture–vug systems therefore still require validation through physical experiments. By contrast, transparent micromodels can directly record gas–water distributions in controllable pore structures. They provide direct evidence for interpreting pore-scale flow processes [23,24,25,26,27].

The present experiments mainly investigate how fractures, vugs, narrow throats, and their connectivity affect gas–water migration, residual-fluid distribution, and electrical response at the pore scale. In actual formations, pore structures are more complex and may evolve because of stress changes, dissolution, diagenesis, and fluid–rock interactions. Future studies should therefore combine micromodel observations with CT, neutron scattering, NMR, acoustic logging, and formation-evaluation data to relate pore-scale mechanisms to in situ pore-size distribution, permeability, and fluid interpretation [10,28,29,30].

The rock electrical response of reservoirs provides a fundamental basis for hydrocarbon exploration, development, and fluid identification. Archie’s equation established an empirical relationship among resistivity, porosity, and water saturation, and it laid the foundation for reservoir evaluation. The Cole–Cole model provides a theoretical basis for analyzing complex impedance and dispersion characteristics in porous media [31]. In complex carbonate reservoirs, strong pore-structure heterogeneity and highly discrete fluid distribution cause conventional rock electrical relationships to differ among pore types; recent numerical and electrical rock-typing studies further show that carbonate electrical parameters are strongly constrained by pore architecture and formation resistivity factor variability [32,33,34,35,36]. Recent digital-rock simulations and experimental/electrical rock-typing studies further demonstrate that pore geometry, rock–fluid interactions, and hydraulic-electric classification can improve saturation and resistivity interpretation in carbonate reservoirs [37,38,39]. However, most rock electrical experiments still focus on bulk core resistivity and average water saturation. This makes it difficult to directly relate the measurements to the spatial distribution of the water phase in pore space. In carbonate reservoirs where fractures, vugs, and narrow throats are jointly developed, resistivity variation often depends on whether the water phase connects main fractures and key pore throats. Average water saturation alone is therefore insufficient for interpreting electrical responses.

Previous studies have improved glass micromodel methods for fractured porous media [23], revealed pore-scale mechanisms of two-phase mass transfer in fracture–matrix systems [24], and summarized the advantages and limitations of micromodels in two-phase flow studies [25]. These studies indicate that pore-scale visualization experiments can link digital structure characterization with macroscopic flow interpretation. However, these studies mainly focused on fluid visualization, and the corresponding electrical response was not measured synchronously.

In this study, an integrated glass-etched micromodel platform for gas–water displacement and electrical measurement was established. First, pore structures were extracted from CT images of real carbonate cores. Three types of glass-etched micromodels were then prepared, including a fracture–vuggy type with large aperture, a fracture–vuggy type with small aperture, and a vuggy type. This design improved the geological representativeness of the micromodels. For flow testing, dyed formation water was injected into the models. The models were first brought to an initial water-saturated state. Gas–water displacement and water–gas displacement were then conducted sequentially to record gas–water distributions, main flow channels, and residual-fluid locations in different pore structures. For electrical measurement, gold-plated electrodes were integrated at the inlet and outlet ends of the etched chip. A constant-flow syringe pump, microscopic imaging system, image acquisition software, and impedance analyzer were combined to achieve synchronous acquisition of fluid images, water saturation, and impedance data. To address this gap, microscopic imaging and impedance measurement were synchronized in CT-derived carbonate micromodels. Each impedance value was matched to the fluid distribution at the same displacement state. This provides a direct experimental basis for relating water-phase connectivity to staged resistivity variation and non-Archie behavior.

## 2. Experiments and Methods

### 2.1. Construction of the Glass-Etched Micromodel Experimental System

**Figure 1:**
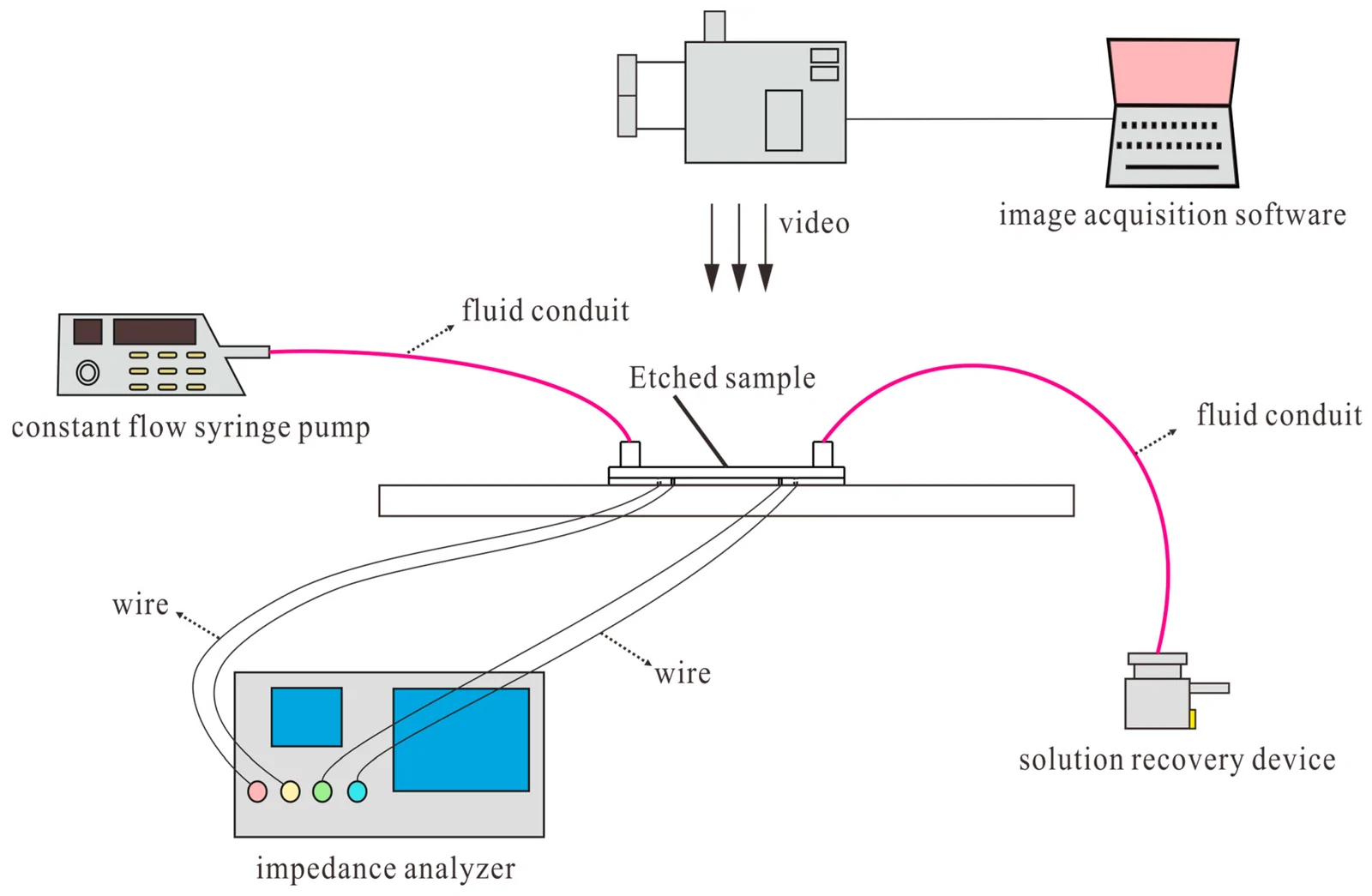
Schematic diagram of the glass-etched micromodel platform for gas–water displacement and synchronous impedance measurement.

An integrated glass-etched micromodel platform for gas–water displacement and electrical measurement was constructed in this study (Figure 1). The platform consists of four subsystems: a constant-rate displacement system, a microscopic imaging system, an impedance measurement system, and an image acquisition system. The system mainly includes an LSP02-1Y constant-flow syringe pump, a HIOKI IM3570 impedance analyzer, a 2802-B video electronic microscope, supporting image acquisition software, and glass-etched samples with integrated conductive electrodes. The overall experimental strategy was to observe the evolution of fluid distribution in the samples in real time through the visualization unit while maintaining a stable displacement flow rate. When the fluid distribution changed significantly, the system synchronously recorded impedance data from the sample, thereby establishing accurate spatiotemporal correspondence between fluid migration behavior and electrical response characteristics.

This design was developed around the experimental objective of “visual displacement–synchronous electrical measurement”. Gold-plated electrodes were integrated at the inlet and outlet ends of the model so that the electrical measurement area was consistent with the main flow area. This arrangement also reduced interference from the electrode structure on fluid migration pathways, thereby improving the reliability of the coupled analysis of fluid distribution and impedance response. The glass samples were 3 cm × 2 cm in size. The etching depth produced in one cycle ranged from 5 to 50 μm. The depth was selected according to the designed pore size. This range was sufficient for typical pores and throats. A greater depth was generally avoided because it could cause uneven channels and wall deformation. For large vugs requiring a depth greater than 50 μm, repeated etching could be used to increase the local depth. A surface gold-plating process on a chromium metal substrate was used for the electrodes to enhance electrical stability. These parameters indicate that the system can effectively balance pore-structure representation accuracy, optical observation clarity, and electrical measurement sensitivity.

From the perspective of system function, this experimental platform performs the core task of simulating gas–water displacement processes in samples with different pore structures. Based on continuous acquisition of image information at different displacement stages, the system enables accurate identification of liquid-phase, gas-phase, and rock-skeleton regions. In addition, impedance data under different water saturation states were completely recorded, providing a data basis for subsequent resistivity calculation, impedance-characteristic analysis, and interpretation of rock electrical parameters.

### 2.2. Design and Fabrication of Glass-Etched Samples

The glass-etched micromodels used in this study did not adopt regular artificial pore networks. Instead, pore structures were extracted from CT images of real carbonate cores and converted into processable two-dimensional etching templates. This design can preserve the main geometric characteristics of fractures, vugs, and pore-throat connectivity under controllable experimental conditions, thereby improving the ability of the micromodels to characterize pore structures in deep complex carbonate reservoirs.

**Figure 2:**
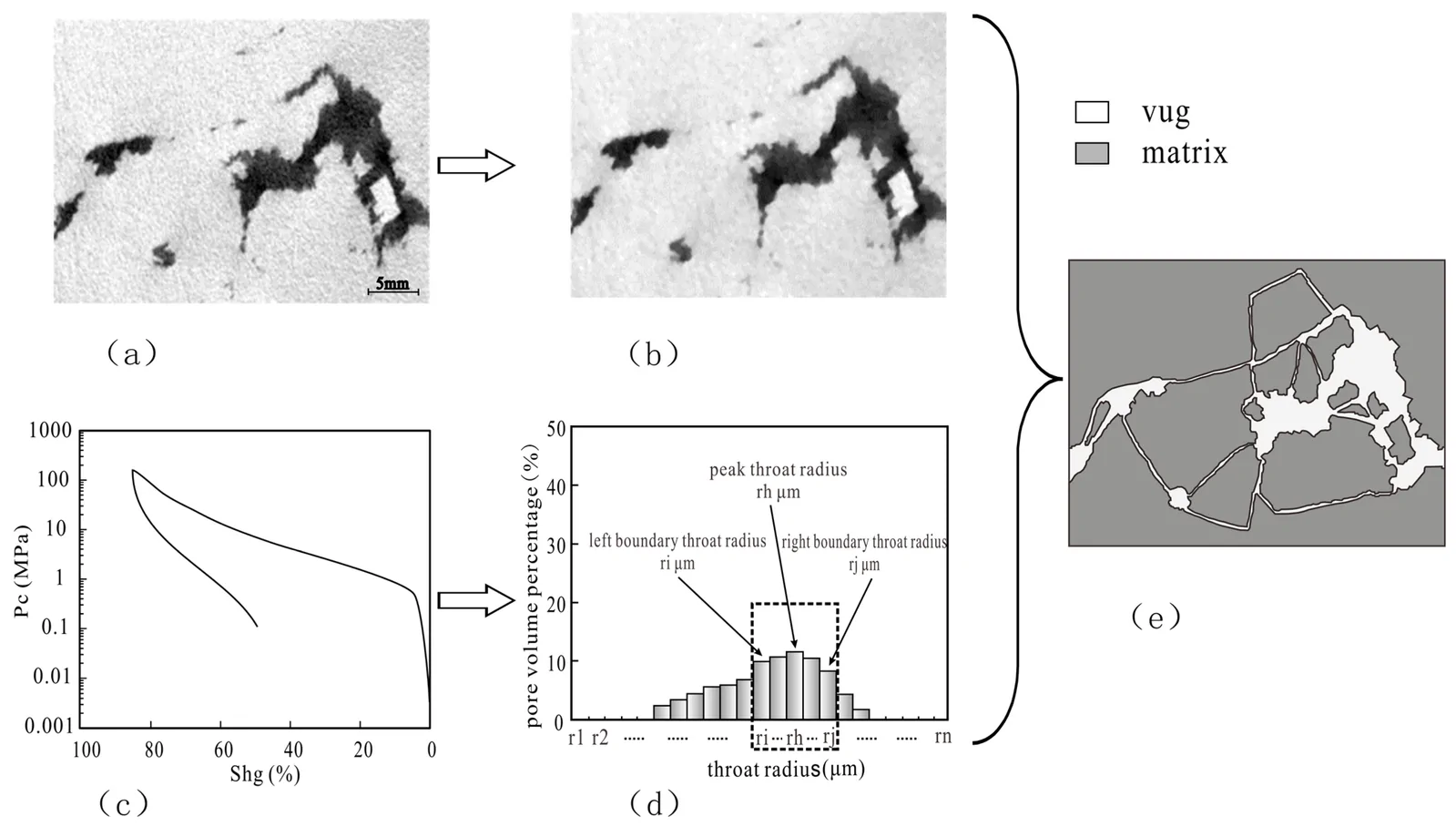
**Workflow of core CT image processing and pore-structure extraction:** (**a**) original CT image; (**b**) filtered image; (**c**) capillary-pressure curve; (**d**) pore-throat radius distribution; (**e**) extracted pore network.

The core pore-structure extraction process mainly included CT image cropping, image denoising, grayscale analysis, threshold segmentation, and pore-network reconstruction (Figure 2). First, the full-diameter core CT scanning data were cropped to select representative regions of interest. Filtering was then applied to reduce image noise and boundary interference, making the grayscale contrast among fractures, vugs, and matrix clearer. On this basis, segmentation thresholds were determined according to peak–valley characteristics in the grayscale histogram, and binary images of pore and fracture regions were extracted. Finally, through pore-throat boundary identification and connectivity analysis, two-dimensional pore-network templates suitable for glass etching were obtained. This workflow effectively preserves major vugs, fracture channels, and narrow throat structures, thereby providing a structural basis for subsequent synchronous gas–water displacement and electrical measurement experiments.

**Figure 3:**
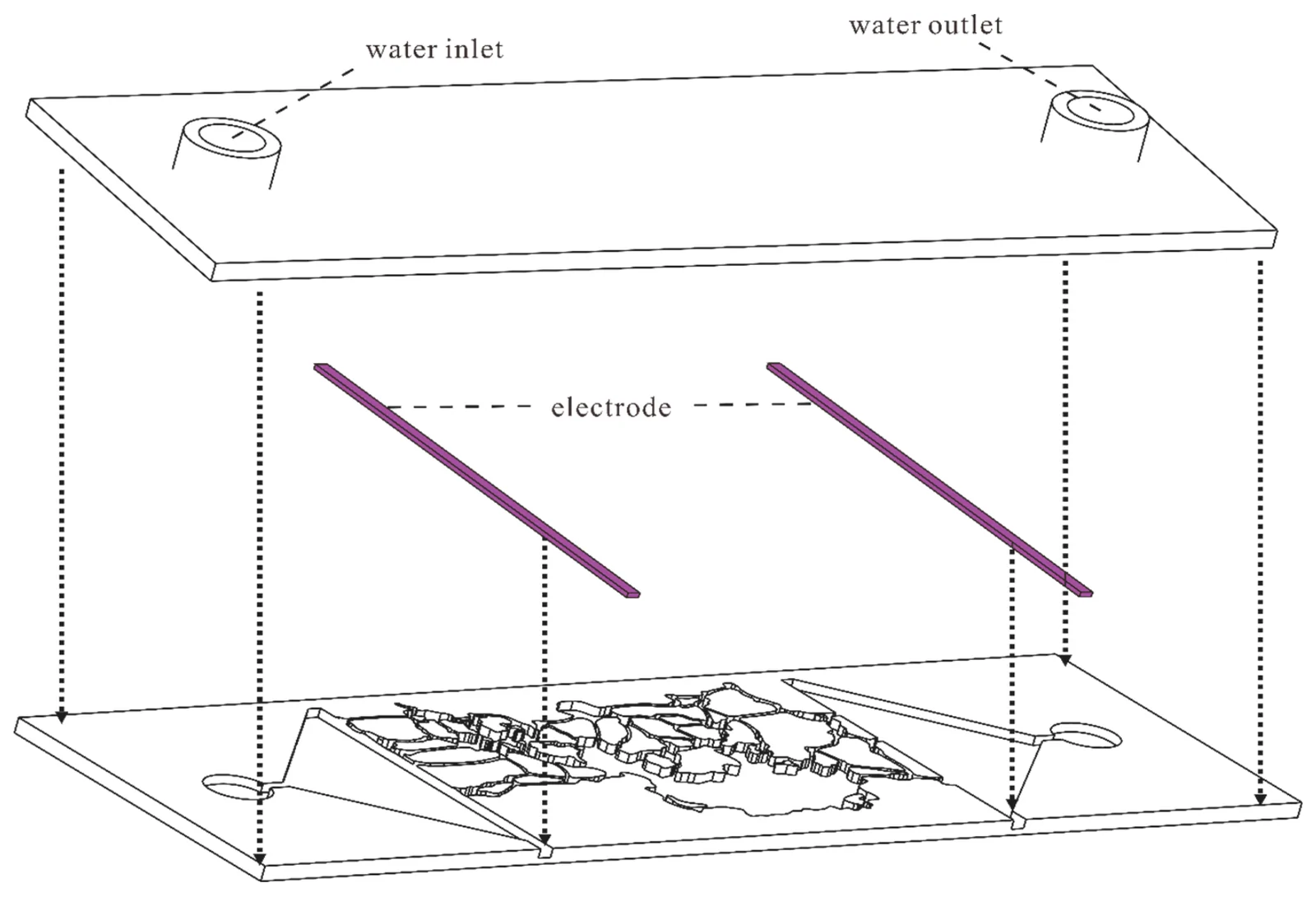
Structural schematic of the electrode-integrated glass-etched micromodel.

Based on the extracted pore-structure templates, chip-scale glass etching was used to transform real-core pore networks into visual micromodels. Each sample consisted of a glass substrate, etched pore network, fluid inlet, fluid outlet, and conductive electrodes (Figure 3). Unlike previous glass-etched models that mostly used regular artificial pore structures and were mainly applied to visual fluid observation, the pore networks in this study were directly derived from the CT-image-based extraction results of real carbonate rocks and were mapped proportionally according to the original pore-throat geometric relationships. Thus, the main connectivity characteristics among fractures, vugs, and narrow throats were well preserved. Fluid inlets and outlets were arranged at both ends of the model to ensure stable entry and discharge of gas and formation water through the pore network. To achieve electrical measurement, metal electrodes were integrated in situ inside the model. The electrodes used a chromium metal substrate with a surface gold-plating process to improve electrical stability and measurement repeatability. This structure allowed the electric-field coverage area to include the main pore network while avoiding obvious obstruction of fluid channels by the electrodes, thereby meeting the experimental requirement for simultaneous flow visualization and impedance measurement.

**Figure 4:**
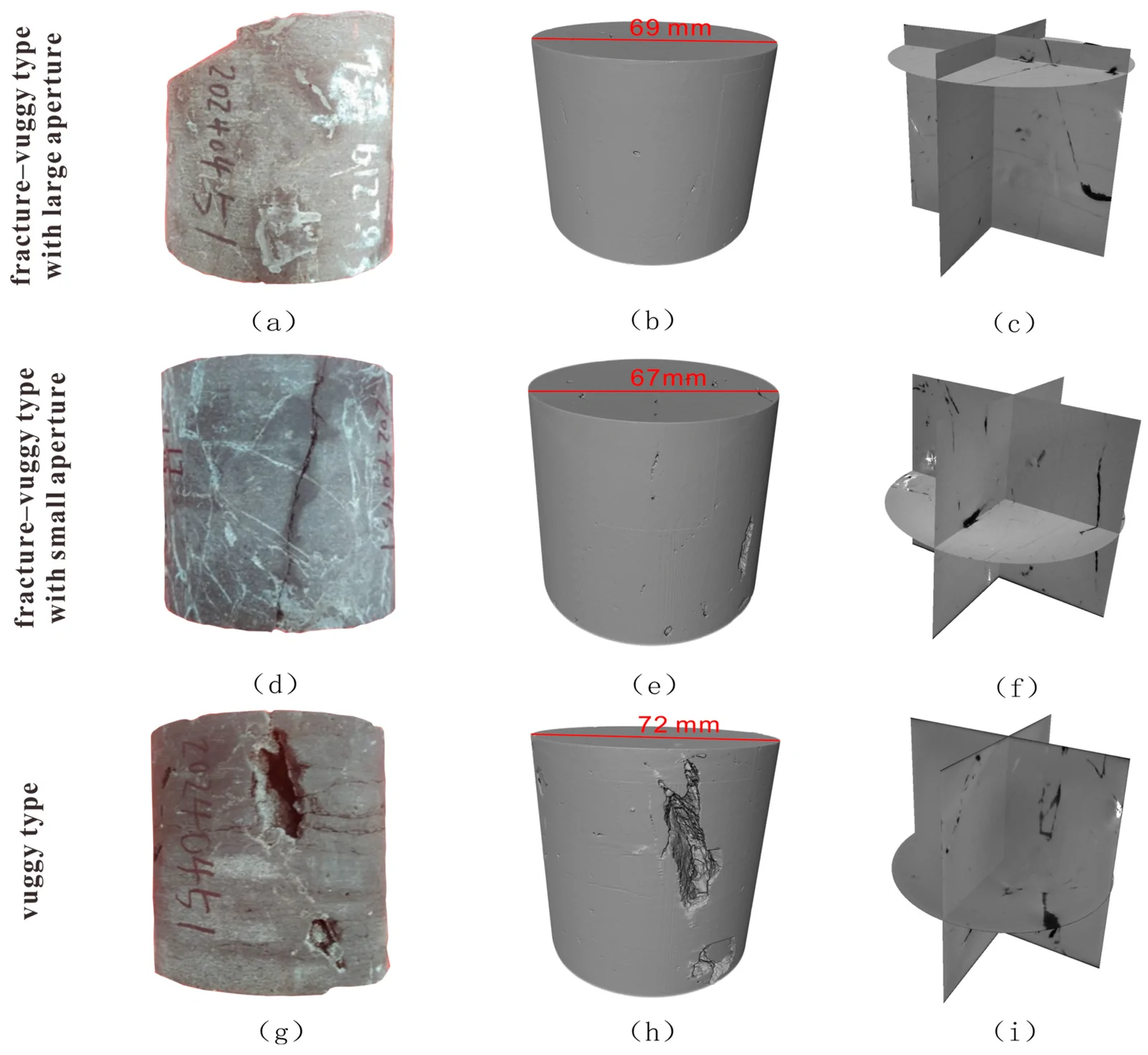
Typical core photographs from Well A: (**a**–**c**) core photograph, CT-based three-dimensional reconstruction, and orthogonal CT slice images of the fracture–vuggy type with large aperture, respectively; (**d**–**f**) corresponding images of the fracture–vuggy type with small aperture; (**g**–**i**) corresponding images of the vuggy type.

Based on typical dolomite core photographs and CT images from Well A, and considering fracture development, vug-distribution characteristics, and pore-throat connectivity patterns, the reservoir pore structures were classified into three types: fracture–vuggy type with large aperture, fracture–vuggy type with small aperture, and vuggy type (Figure 4). (1) In the fracture–vuggy type with large aperture, fractures and filled veins are relatively well developed inside the core, and local dissolution vugs occur. The pore space shows strong directionality and throughgoing connectivity. The two-dimensional areal porosity is 14.53%. This structure is suitable for characterizing rapid gas–water two-phase migration, preferential occupation of main channels, and abrupt changes in the conductive network under large-scale fracture control. (2) The fracture–vuggy type with small aperture uses continuous inclined fractures as the main skeleton for fluid migration and contains a small number of fine fractures and local vugs. Compared with the fracture–vuggy type with large aperture, this structure has markedly smaller fracture aperture and main-channel scale, and the pore-throat connectivity is relatively limited. Its two-dimensional areal porosity is 13.06%. This structure can be used to analyze displacement-front propagation, residual-fluid trapping, and resistivity-response variation after fracture channels become narrower. (3) The vuggy-type core is relatively intact overall, without developed macroscopic fractures. Its pore space is mainly contributed by local dissolution vugs and weakly connected pore throats. Its two-dimensional areal porosity is 9.40%. This structure reflects bypass flow, local trapping, and progressive conductive-network variation under dispersed vug-connected conditions.

The design of these three types of glass-etched models mainly considered differences in pore-structure type and pore-throat scale. The fracture–vuggy type with large aperture highlights the control of large-scale fractures on preferential channels. The fracture–vuggy type with small aperture reflects transitional characteristics jointly controlled by narrow fractures and local vugs. The vuggy type emphasizes heterogeneous flow behavior under dispersed vug-connected conditions. Gas–water displacement and water–gas displacement experiments conducted on the three model types can further reveal the controlling mechanisms of different pore structures on two-phase fluid migration, residual-fluid occurrence, and resistivity response. The glass-etched micromodel is a two-dimensional representation of a three-dimensional pore network. During this conversion, out-of-plane connections are removed. This may change pore connectivity, flow-path tortuosity, fluid trapping, and current pathways. The CT-derived templates nevertheless retain the main fracture directions, vug distribution, pore-throat sizes, and in-plane connectivity. All three models were prepared and tested using the same fabrication method, fluids, boundary conditions, electrode arrangement, and experimental procedure. The dimensional constraint was therefore consistent among the models. The results are used to compare their relative flow and electrical responses rather than to provide absolute three-dimensional reservoir parameters. In addition, the planar model allows each impedance value to be matched directly with the fluid distribution at the same displacement state.

### 2.3. Gas–Water Two-Phase Displacement Scheme and Electrical Measurement Procedure

The gas–water two-phase displacement experiment included three stages: initial water saturation, gas–water displacement, and water–gas displacement. These stages correspond to the initial water-saturated state of the reservoir, gas-induced water drainage during natural gas accumulation, and gas–water redistribution caused by water invasion during late-stage development, respectively. Before the experiment, synthetic formation water was prepared according to formation-water salinity and ionic composition, and dye was added to enhance water-phase distinguishability. The glass-etched model was then connected to the constant-flow syringe pump, impedance analyzer, and microscopic imaging system, and the fluid pipeline, electrode interface, and image acquisition state were checked. Dyed formation water was injected at 0.001 mL/min until no visible gas bubbles remained in the pore network. The model was then considered fully water-saturated, and the initial water saturation was recorded as *S_w_*_0_ = 100%.

**Figure 5:**
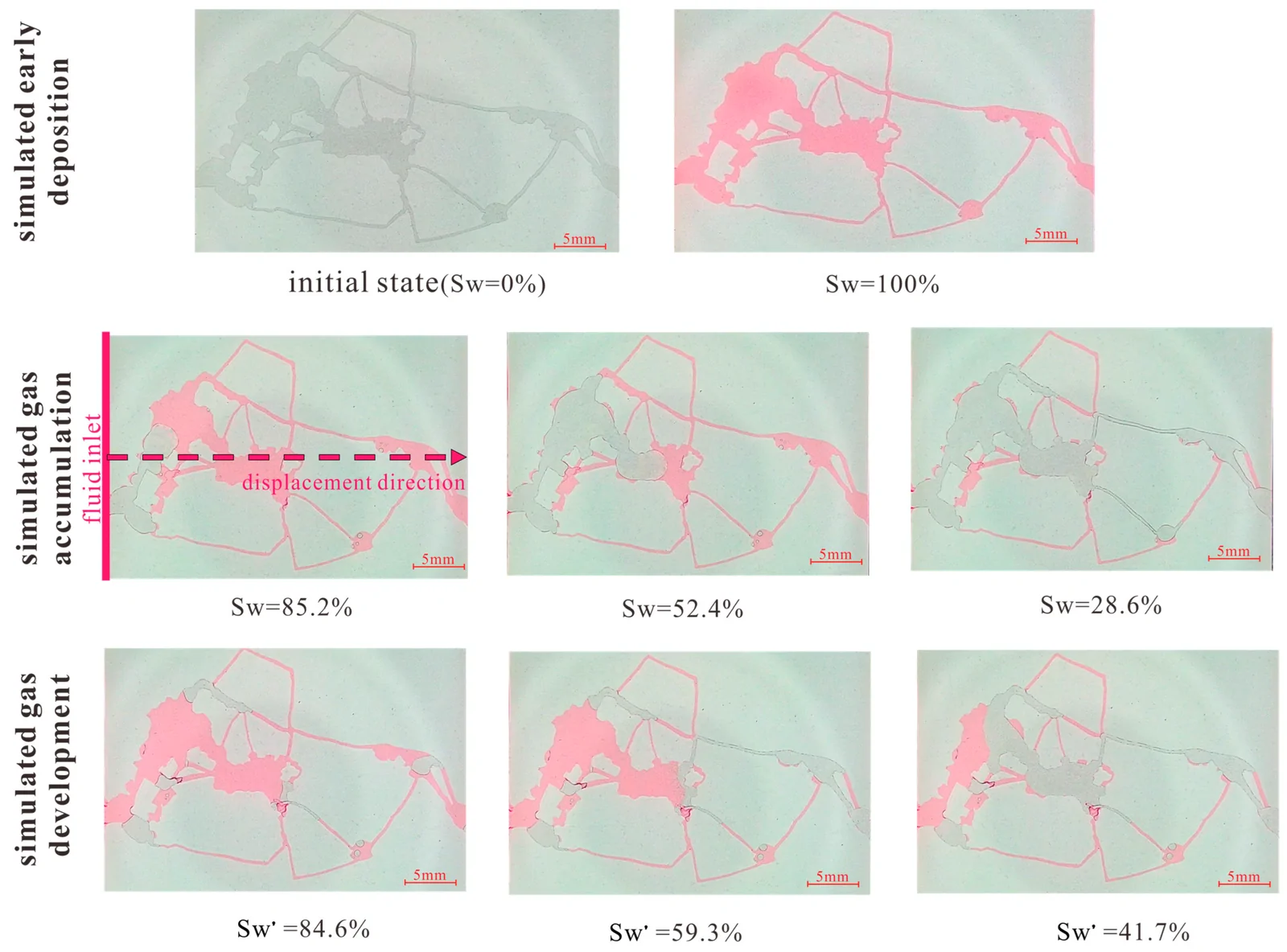
Experimental workflow of gas–water displacement and water–gas displacement and representative recorded water-saturation states. The pink regions represent dyed formation water, whereas the light-gray regions represent gas-filled pore space. The magenta dashed arrow indicates the displacement direction.

During the gas–water displacement stage, air was injected into the model at a nominal flow rate of 0.001 mL/min. The flow rate was kept constant throughout this stage. Air gradually displaced the dyed formation water along the pore network. The microscopic imaging system recorded gas–water interface migration and residual-water distribution changes in real time. When the fluid distribution changed markedly, the corresponding images were acquired, and impedance data were synchronously recorded. As gas invasion continued, the water saturation gradually decreased from *S_w_*_0_ to *S_w_*_1_, *S_w_*_2_, and *S_wn_*, and finally approached the irreducible water state (Figure 5). Air was selected as the gas phase to ensure safe and stable visualization and electrical measurements. The experiments were conducted at 25 °C and atmospheric pressure (1 atm). The flow rate was maintained at 0.001 mL/min. Differences between air and natural gas may affect quantitative results under reservoir conditions, but their influence on the relative trends obtained under the same experimental conditions is expected to be limited.

During the water–gas displacement stage, dyed formation water was reinjected at 0.001 mL/min. The flow rate was kept constant throughout this stage. The injected water gradually displaced the gas in the pore space. This stage focused on observing water-phase re-occupation of the main channel, residual-gas formation, and residual-gas trapping in vugs, narrow throats, and poorly connected regions. Microscopic images and impedance data were also synchronously acquired when the fluid distribution changed significantly, allowing the water-phase recovery process to correspond one-to-one with the electrical response.

Microscopic images show clear differences among dyed formation water, gas-phase regions, and the glass skeleton, which can be used for subsequent image segmentation and water saturation calculation (Figure 5). During gas–water displacement, gas preferentially entered fractures, large pore throats, and preferential channels with stronger connectivity, whereas the water phase mainly remained in vug corners, fine fractures, and dead-end regions. During water–gas displacement, the water phase re-entered the model along preferential channels, but part of the gas remained trapped inside vugs and at the ends of narrow throats. This phenomenon indicates that the gas–water displacement process has obvious path dependence, and that fluid distribution is jointly controlled by pore structure and channel connectivity.

**Figure 6:**
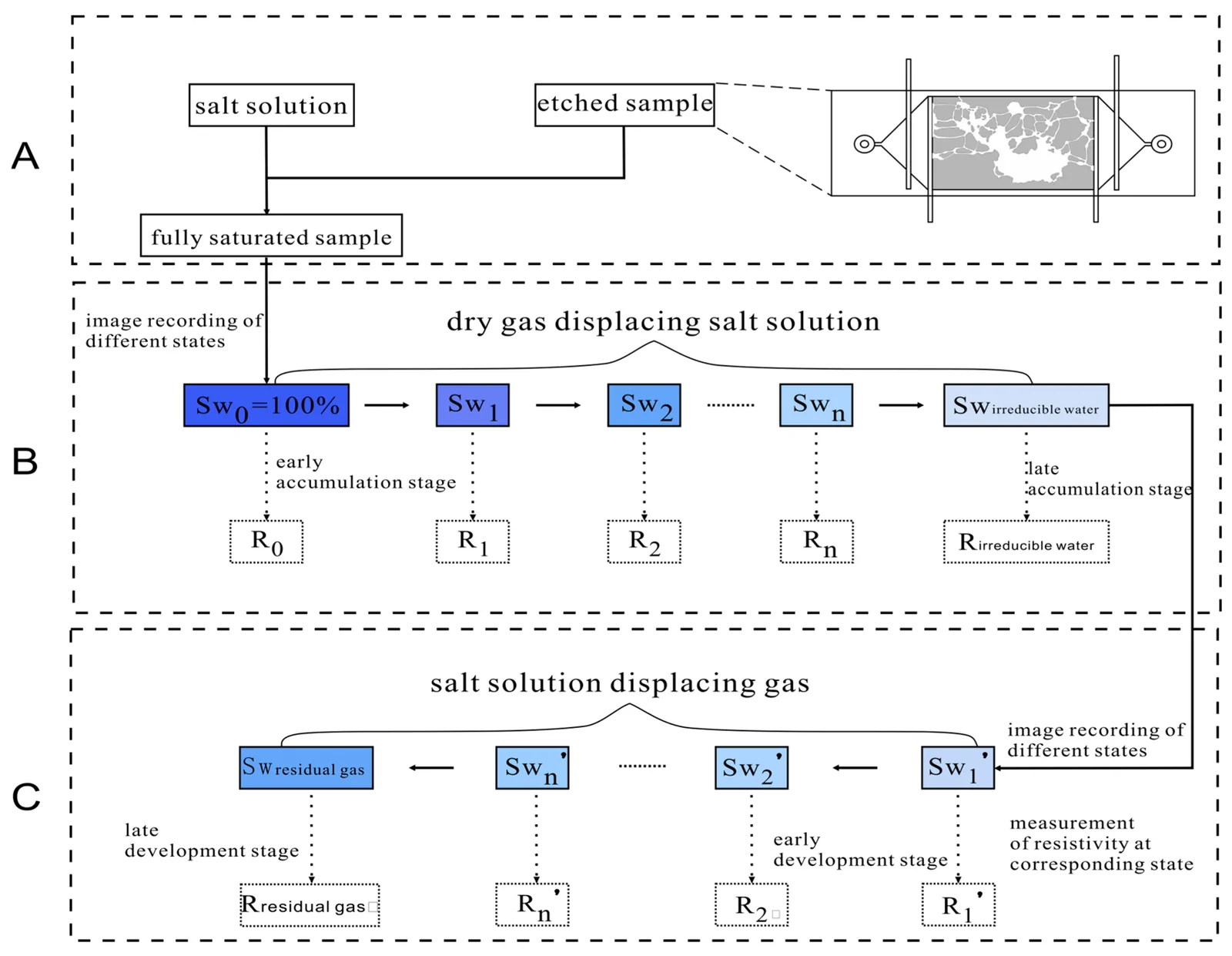
Representative fluid distributions and water-saturation variations during gas–water displacement and water–gas displacement. (**A**) preparation of the glass-etched micromodel and complete saturation with dyed formation water; (**B**) gas–water displacement, showing the recorded water-saturation and resistance states from the fully water-saturated state to the irreducible-water state; and (**C**) water–gas displacement, showing the recorded water-saturation and resistance states during water reinjection until the residual-gas state was reached.

The staged displacement scheme can continuously record gas invasion and water-phase back-displacement in the same glass-etched model, thereby reflecting the entry, trapping, and redistribution characteristics of two-phase fluids in different pore structures. In Figure 6A, the etched model was fully saturated with dyed formation water. In Figure 6B, air was injected to displace the formation water. Water saturation decreased from Sw_0_ = 100% to the irreducible water state. The corresponding resistivity values were recorded as R0, R1, R2, …, Rn, and Rirreducible water. In Figure 6C, formation water was reinjected to displace the air. Water saturation gradually increased until the residual-gas state was reached. The corresponding resistivity values were recorded as R1′, R2′, …, Rn′, and Rresidual gas. This procedure allowed the fluid distribution, water saturation, and resistivity to be compared at the same displacement state.

Electrical measurement was performed using the two-electrode method. The gold-plated electrodes at both ends of the model were connected to the impedance analyzer to measure impedance responses under different water saturation states. Each recorded state was measured multiple times and averaged to reduce the effects of instrumental fluctuation and contact error. Impedance measurement and microscopic imaging were conducted synchronously, so each set of electrical data corresponded to a definite fluid distribution state and water saturation.

Before selecting the operating frequency, three continuous frequency sweeps from 4 Hz to 5 MHz were performed under the same fluid-distribution condition. The measured resistance decreased rapidly in the low-frequency range, indicating a relatively strong electrode-polarization effect. The resistance gradually approached a stable value near 1 kHz, where the influence of electrode polarization was substantially reduced. Therefore, an AC frequency of 1 kHz was selected for the subsequent resistance measurements. Near 1 kHz, the coefficient of variation for three repeated measurements was approximately 0.26%, indicating good repeatability. The electrodes consisted of a chromium substrate coated with a gold layer. Before each experiment, the electrode surfaces were polished and cleaned to remove possible surface contamination. The contact pressure was then increased to enlarge the effective contact area. The electrode positions, wire connections, and applied pressure were kept constant during the measurements to minimize variations in contact resistance.

This experimental procedure achieved synchronous acquisition of gas–water displacement, fluid distribution, and impedance response. Based on this procedure, differences in main flow channels, residual-fluid distribution, and resistivity response can be further analyzed for the fracture–vuggy type with large aperture, fracture–vuggy type with small aperture, and vuggy type.

### 2.4. Data Processing

The data processing in this study mainly included two parts: image segmentation and water saturation calculation, and impedance data organization and rock electrical parameter analysis. First, during image processing, the acquired microscopic images were cropped and subjected to grayscale correction to remove invalid boundaries and background interference. Threshold segmentation was then performed according to the color and grayscale differences among dyed formation water, gas-phase regions, and the glass skeleton, and the water-phase area and total pore area within the effective pore region were extracted. For regions with a uniform etching depth, water saturation was calculated as × *S_w_* = (*A_w_*/*A_p_*) × 100%, where *A_w_* is the water-filled area and *A*_p_ is the total pore area. For regions with different etching depths, water saturation was calculated as *S_w_* = [Σ(*A_w,i_* × *h_i_*)/Σ(*A_p,i_ h_i_*)] × 100%, where *h_i_* is the etching depth of region *i*. This depth-weighted equation reduces to the two-dimensional area ratio when *h_i_* is constant.

Impedance data processing was synchronized with image analysis. The impedance data acquired under each displacement state were matched with the corresponding microscopic images, and repeated measurements were averaged to reduce the influence of instrumental fluctuation and electrode contact error. The impedance results were then organized in combination with the geometric dimensions, electrode arrangement, and measurement parameters of the glass-etched model, yielding resistivity responses under different water saturation conditions. After processing, each data point could be associated with a definite fluid distribution state, avoiding the difficulty of explaining microscopic causes when resistivity curves are analyzed alone.

In the analysis of rock electrical parameters, Archie’s theory was used as the basic reference, with emphasis on the relationship between the resistivity index and water saturation. The resistivity index can be expressed as *I* = *R_t_*/*R*_0_, where *R_t_* is the resistivity at a given water saturation state, and *R*_0_ is the resistivity at full water saturation. In conventional homogeneous porous media, I and Sw usually exhibit a relatively stable power-law relationship. However, fractures, vugs, and narrow throats in complex carbonate reservoirs change the distribution pattern of the continuous water-phase network, causing the rock electrical response to exhibit obvious heterogeneous characteristics.

Resistivity variation should be interpreted on the basis of water saturation together with image-segmentation results and impedance measurement data. For the fracture–vuggy type with large aperture, fracture–vuggy type with small aperture, and vuggy type, the key comparisons focused on whether the water phase occupied preferential channels, whether the main conductive pathways were interrupted, and whether residual water was retained in vug corners or fine-fracture regions. This analysis further identifies the microscopic controlling factors responsible for segmented resistivity variation and deviation of rock electrical parameters from the Archie relationship.

Overall, the data processing workflow in this study achieved continuous conversion from images to water saturation, from impedance to resistivity, and from fluid distribution to rock electrical parameters. This workflow not only preserves the visualization advantage of glass-etched micromodel experiments, but also provides the electrical response with a clear interpretive basis in pore structure and fluid distribution. It therefore supports subsequent discussion of staged resistivity responses and non-Archie behavior under different pore structures.

## 3. Results and Discussion

### 3.1. Gas–Water Two-Phase Flow Characteristics Under Different Pore Structures

**Figure 7:**
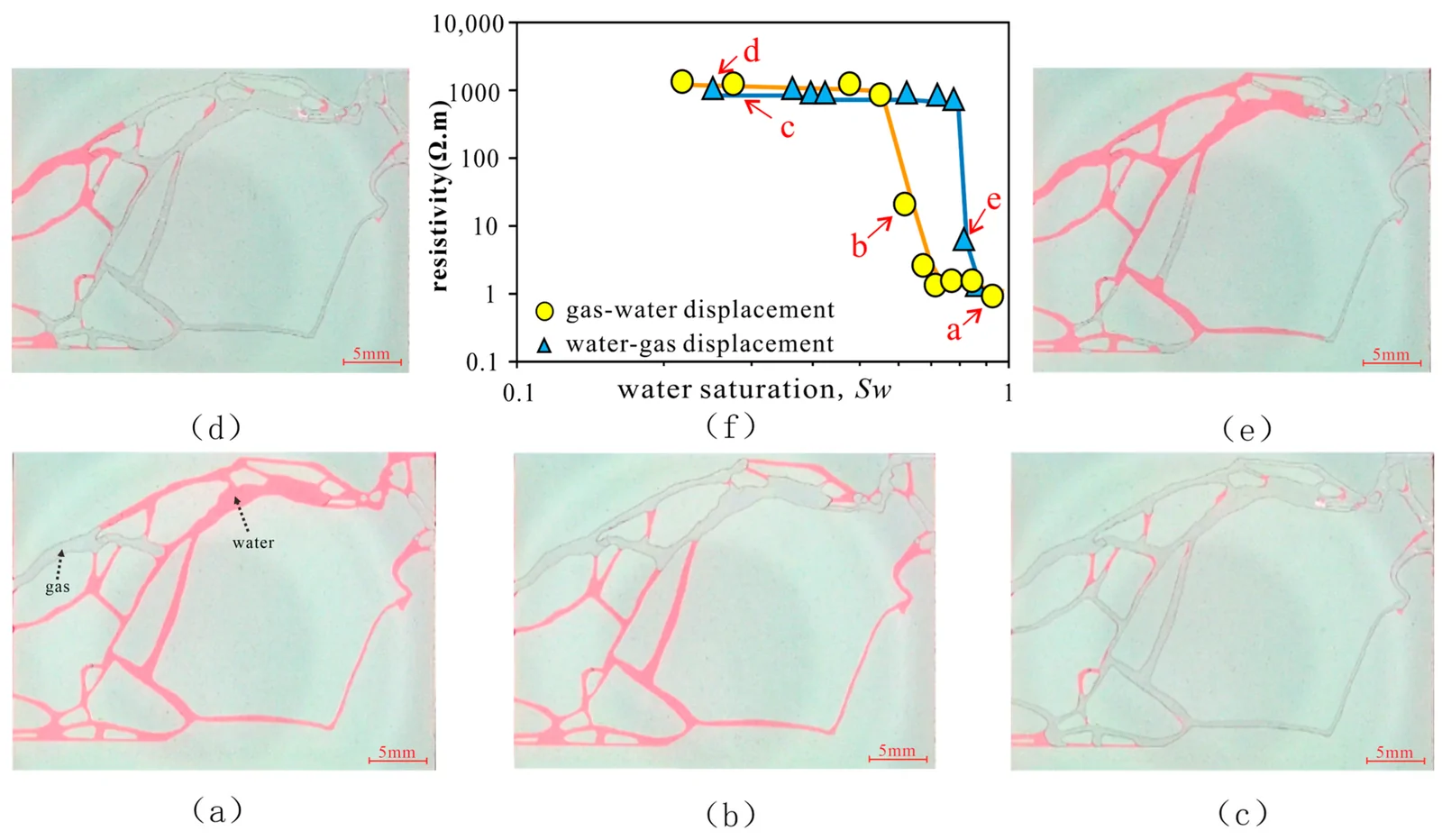
Fluid distribution and resistivity-response stages during gas–water displacement in the fracture–vuggy type with large aperture: (**a**–**c**) representative fluid-distribution states during gas–water displacement; (**d**,**e**) representative fluid-distribution state during water–gas displacement; (**f**) staged resistivity–water saturation response.

**Figure 8:**
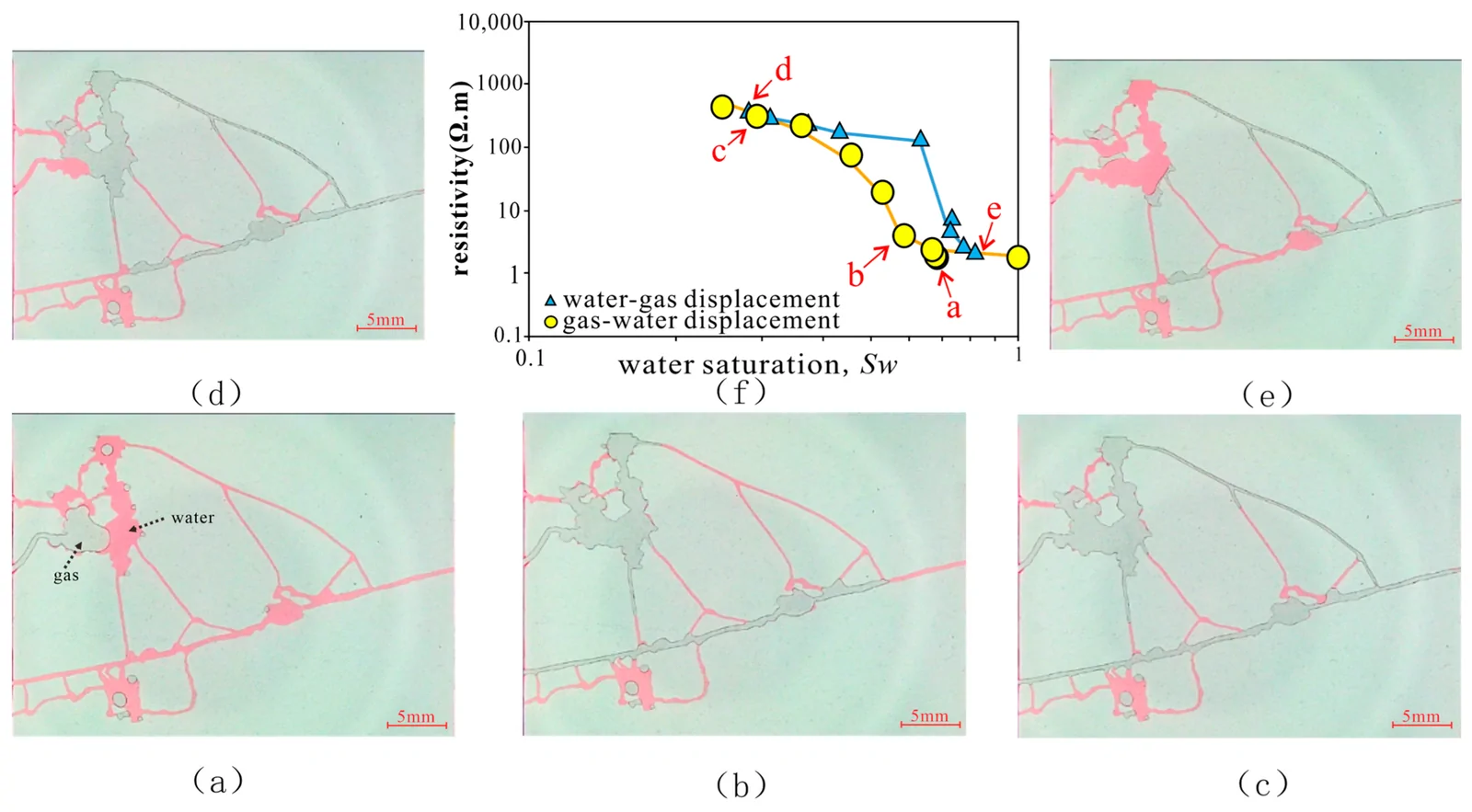
Fluid distribution and resistivity-response stages during gas–water displacement in the fracture–vuggy type with small aperture: (**a**–**c**) representative fluid-distribution states during gas–water displacement; (**d**,**e**) representative fluid-distribution state during water–gas displacement; (**f**) staged resistivity–water saturation response.

**Figure 9:**
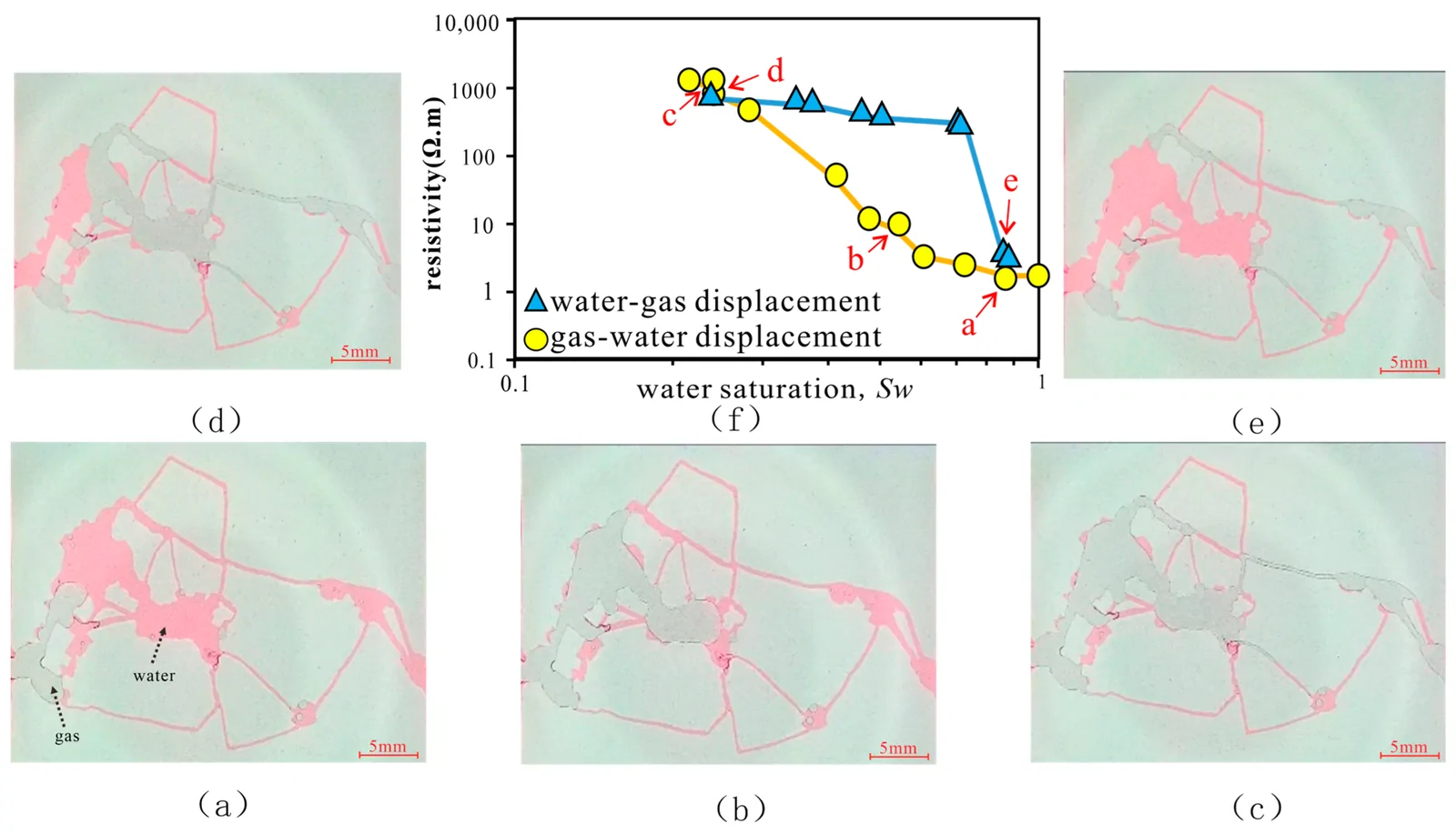
Fluid distribution and resistivity-response stages during gas–water displacement in the vuggy type: (**a**–**c**) representative fluid-distribution states during gas–water displacement; (**d**,**e**) representative fluid-distribution state during water–gas displacement; (**f**) staged resistivity–water saturation response.

Glass-etched models with different pore structures showed obvious differences during gas–water two-phase displacement. Combining the core photographs, CT reconstruction results, and microscopic displacement images described above, the three structural types gradually transition from strong fracture control to dispersed-vug connectivity control (Figure 4, Figure 7, Figure 8 and Figure 9). As fracture aperture decreases and vug connectivity weakens, the dominant fluid-flow channel gradually changes from a concentrated throughgoing type to a tortuous bypass-flow type, and the distribution range of residual fluids also expands.

In the fracture–vuggy type with large aperture, wide fractures and connected vugs jointly form the main flow channels. During gas–water displacement, gas rapidly invades along large-aperture fractures, and the water phase in the main channels is quickly displaced, producing a relatively concentrated displacement front. Because main-channel control is strong, fine fractures, vug corners, and local dead-end regions outside the main flow areas are more likely to retain residual water. During water–gas displacement, the water phase still mainly back-displaces gas along the original preferential channels, but part of the gas remains trapped at vug margins and in local low-disturbance zones. This structure shows typical preferential-channel-dominated characteristics. Fluid migration efficiency is relatively high, but local residual fluids remain difficult to displace completely.

The fracture–vuggy type with small aperture still has a relatively clear connected fracture skeleton, but the main-channel width and connectivity scale are markedly smaller than those of the fracture–vuggy type with large aperture. During gas–water displacement, gas mainly advances along inclined fractures and locally splits at fracture–vug connections. Because the channels are narrowed, capillary retardation and local trapping are enhanced. The water phase no longer remains only in vug corners, but forms more discrete water clusters in fine fractures, narrow throats, and vug-connection zones. During water–gas displacement, the water phase can reoccupy part of the main channel, but local gas remains trapped in weakly connected vug units. This structure reflects transitional characteristics controlled jointly by preferential channels and local trapping.

The vuggy type lacks continuous large-scale fractures, and fluid migration is mainly controlled by narrow throats and local connectivity among vugs. During gas–water displacement, gas cannot rapidly break through along a single channel; instead, it gradually splits and bypasses among multiple vug units. The displacement front is more dispersed, and residual water is easily formed inside local vugs, in angular corner regions, and at poorly connected throats. During water–gas displacement, water-phase back-displacement also shows strong heterogeneity, and part of the gas is captured by closed or semi-closed vugs to form local residual gas. This structure has the most discrete fluid distribution, and the displacement process is more easily constrained by pore-throat scale differences and local connectivity.

**Figure 10:**
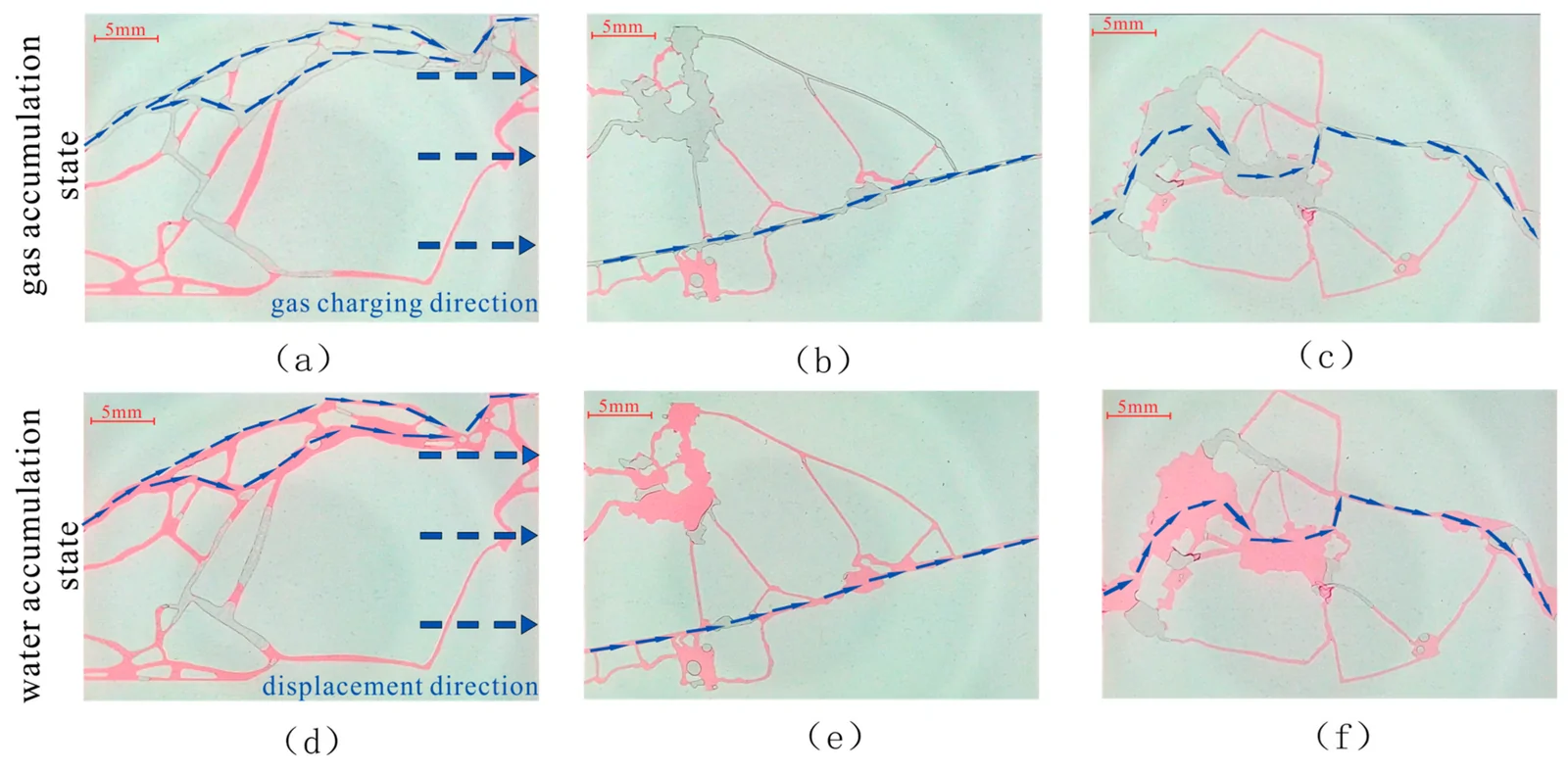
Main flow channels and residual-fluid distributions during gas–water two-phase displacement in different pore structures: (**a**–**c**) gas–water displacement in the fracture–vuggy type with large aperture, fracture–vuggy type with small aperture, and vuggy type, respectively, showing the main gas-invasion channels and residual-water distributions; (**d**–**f**) corresponding water–gas displacement states, showing the main water-invasion channels and residual-gas distributions. The blue dashed lines indicate the principal flow paths, the blue arrows indicate the displacement directions, and the pink regions represent dyed formation water.

The extracted main flow channels further demonstrate the control of pore structure on two-phase flow pathways (Figure 10). In the fracture–vuggy type with large aperture, the fluid channels are concentrated and strongly throughgoing, and irreducible water is mainly distributed in small corners and non-main-flow regions. In the fracture–vuggy type with small aperture, the main channel is still identifiable, but channel convergence enlarges the distribution range of residual fluids. In the vuggy type, the fluid pathways are more tortuous, local bypass flow and side flow are more obvious, and irreducible fluids are widely retained in vug corners and weakly connected zones.

Overall, pore structure is the dominant factor controlling gas–water two-phase displacement behavior. The larger the fracture aperture, the more readily fluids advance rapidly along preferential channels and the more concentrated the displacement process becomes. The more dispersed the vug connectivity, the more likely the fluid front is to undergo bypass flow, flow splitting, and local trapping. These differences provide direct pore-scale fluid-mechanical evidence for subsequently interpreting staged resistivity responses and deviations of rock electrical parameters from the Archie relationship.

### 3.2. Evolution of Fluid Distribution and Resistivity Response

**Figure 11:**
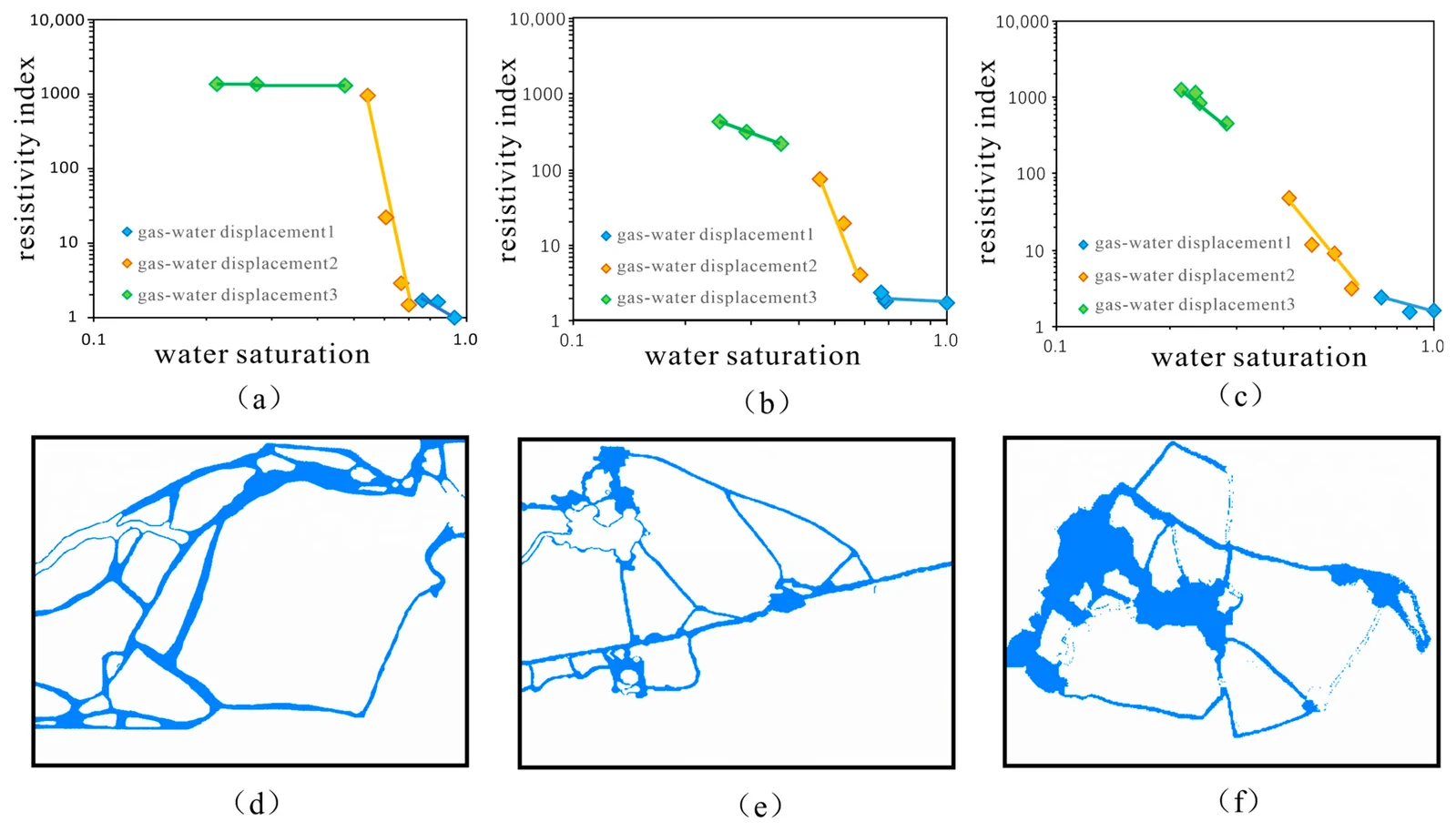
Staged resistivity responses and evolution of effective conductive pathways in different pore structures. Curves (**a**–**c**) show differences in resistivity responses among the three pore structures during gas–water displacement. Panels (**d**–**f**) correspond to pore-channel extraction results from the glass-etched models.

After clarifying the gas–water displacement characteristics of different pore structures, the microscopic images were further correlated with the impedance measurement results. The experimental results show good synchrony between water saturation variation and resistivity response. During gas–water displacement, as the water phase was gradually replaced by gas, water saturation decreased and overall resistivity increased. During water–gas displacement, as the water phase re-entered the pore network, water saturation increased and overall resistivity decreased (Figure 11). Combined with the channel-extraction results for different pore structures, the differences in resistivity response are closely related to the degree of concentration, throughgoing connectivity, and dispersed connectivity characteristics of pore channels.

The resistivity response during gas–water displacement can be divided into three stages: slow increase, rapid increase, and renewed slow increase. At the initial displacement stage, gas begins to enter the pore network, but the continuous water phase still maintains good connectivity. The main conductive pathways have not yet been clearly disrupted, so resistivity increases only slowly. As gas further occupies fractures, large pore throats, or key connected segments, the originally continuous water-phase network is cut off, electrical conductivity capacity decreases rapidly, and resistivity enters the rapid-increase stage. At the late displacement stage, the model mainly contains irreducible water, water film, and local residual water. The conductive network has become highly discrete, and the increase in resistivity slows again.

The water–gas displacement stage exhibits the opposite electrical response process. At the early stage of water-phase re-entry into the pore network, only local conductive pathways can be restored, and resistivity decreases slowly. As the water phase gradually reconnects the main channel, the conductive network is rapidly restored and resistivity decreases significantly. In the late stage, part of the gas remains trapped in vug corners, narrow throats, and poorly connected regions. The degree of water-phase connectivity tends to stabilize, and the magnitude of resistivity decrease gradually diminishes. This process indicates that water–gas displacement is not simply the reverse of gas–water displacement, but is jointly controlled by residual-gas trapping and pore-structure heterogeneity.

The rate of resistivity variation differs markedly among different pore structures. In the fracture–vuggy type with large aperture, fluid and conductive pathways are highly dependent on wide fractures and their connected vugs. Once the main conductive pathway is occupied by gas, the continuous water-phase network is disrupted within a short time, and resistivity rises rapidly. The fracture–vuggy type with small aperture still shows main-channel control, but narrow fractures, fine throats, and local vugs can provide certain alternative conductive pathways; therefore, the resistivity-increase rate is relatively weakened. In the vuggy type, conductive pathways are more dispersed. After gas invasion, multiple weakly connected channels gradually fail, and resistivity changes more gently.

Thus, the key factor controlling the resistivity response is not a single water saturation value, but the spatial connectivity of the water phase in the pore network. Even when water saturation values are similar, the model can still maintain strong electrical conductivity capacity if the water phase is mainly distributed in throughgoing fractures and main pore throats. If the water phase mainly remains in isolated vugs, corner regions, or water-film states, the overall resistivity increases significantly. Therefore, the staged variation in the resistivity curve essentially records the dynamic process in which the continuous water-phase conductive network transitions from connected to disconnected and then from disconnected to reconnected.

Combining image and electrical data indicates that the fracture–vuggy type with large aperture has a more abrupt resistivity response, the fracture–vuggy type with small aperture shows a moderate degree of nonlinear variation, and the vuggy type exhibits more obvious progressive evolution. This difference indicates that resistivity responses in complex carbonate reservoirs are jointly controlled by fracture development, pore-throat connectivity, and the spatial distribution of the water phase. Integrated analysis of visualized fluid distributions and impedance measurement results helps identify the water-invasion process, remaining-gas trapping locations, and connectivity-state changes in main conductive pathways at the pore scale.

### 3.3. Variation Characteristics and Controlling Factors of Rock Electrical Parameters

Based on the staged resistivity response, analysis of rock electrical parameters can further reveal the control of different pore structures on conductive behavior. The experimental results show that all three pore structures exhibited obvious non-Archie characteristics during gas–water displacement. The resistivity index and water saturation did not maintain a stable single power-law relationship. This phenomenon indicates that the rock electrical response in complex carbonate reservoirs is controlled not only by water saturation, but also closely related to the connectivity state of the water phase in the pore network.

For the fracture–vuggy type with large aperture, the rock electrical parameter during the three stages of gas–water displacement, namely the saturation exponent, n, was 2.62, 13.10, and 6.77, respectively, and the overall n value was 7.26. The n value increased significantly in the second stage, indicating that when gas occupied wide fractures and main pore throats, the continuous water-phase conductive network was rapidly cut off, and resistivity produced an anomalously amplified response to changes in water saturation. In this structure, both fluid migration and conductive processes are highly dependent on a small number of preferential channels. Therefore, once the main channel loses connection, the rock electrical response shows strong abruptness.

For the fracture–vuggy type with small aperture, the n values in the three stages were 4.36, 7.24, and 5.18, respectively, and the overall n value was 6.36. Compared with the fracture–vuggy type with large aperture, its second-stage n value was lower, indicating that narrow fractures and local vugs can buffer the rapid disconnection of the conductive network to some extent. This structure is still controlled by the main fracture, but fine fractures, narrow throats, and local vugs provide partial alternative conductive pathways, reducing the abruptness of the resistivity response.

For the vuggy type, the n values in the three stages were 7.25, 9.84, and 5.37, respectively, and the overall n value was 5.39. This structure lacks continuous large-scale fractures, and conductive pathways are mainly controlled by local connectivity among vugs. Although the overall resistivity variation is relatively gentle, failure of key narrow throats or locally connected units within certain water saturation intervals can still cause a significant increase in n. This indicates that the rock-electrical nonlinearity of the vuggy type does not originate from an abrupt change in a single main fracture, but is jointly caused by the progressive disconnection of multiple local conductive pathways.

As shown in Figure 7, Figure 8, Figure 9 and Figure 11, the contrasting electrical responses of the three pore structures cannot be explained solely by the amount of pore space. The two-dimensional areal porosities of the fracture–vuggy type with large aperture, fracture–vuggy type with small aperture, and vuggy type were 14.53%, 13.06%, and 9.40%, respectively. In the fracture–vuggy type with large aperture, water saturation decreased from 69% to 55% during disconnection of the main water-phase conductive network. Over the same interval, resistivity increased sharply from 3 to 900 Ω·m. Thus, a decrease of 14 percentage points in water saturation produced a 300-fold increase in resistivity. This response indicates rapid disconnection of the water-filled main fractures.

In the fracture–vuggy type with small aperture, water saturation decreased from 63% to 48%, while resistivity increased from 4 to 75 Ω·m. The saturation exponent in the second stage was 7.24. Narrow fractures and local pore throats maintained alternative conductive pathways, resulting in a more gradual resistivity increase. In the vuggy type, water saturation decreased from 61% to 43%, while resistivity increased from 3 to 50 Ω·m. As water saturation further decreased to 22%, resistivity gradually increased to 1.3 × 10^3^ Ω·m. The saturation exponent in the second stage was 9.84. This gradual increase indicates sequential disconnection of several weakly connected pore-throat pathways rather than abrupt loss of a single dominant fracture.

These results show that similar decreases in average water saturation can produce different resistivity responses. The fracture–vuggy type with large aperture had the highest two-dimensional areal porosity, but electrical conduction was concentrated in a few well-connected fractures. Gas invasion of these fractures rapidly disrupted the continuity of the water-phase conductive network. Water remaining in isolated vugs and vug corners could not maintain a continuous conductive pathway. Therefore, the observed non-Archie behavior was controlled mainly by the topology and continuity of the water-filled network rather than by porosity or average water saturation alone.

In terms of the overall n value, the three structures follow the order fracture–vuggy type with large aperture > fracture–vuggy type with small aperture > vuggy type. This trend is consistent with the main-flow-channel extraction results discussed above (Figure 10 and Figure 11). The more concentrated the pore structure, the more prominent the preferential conductive pathway, the more easily the resistivity response is controlled by a small number of key channels, and the more readily the rock electrical relationship deviates from the classical Archie model. Conversely, when fluid and conductive pathways are more dispersed, the disruption of the conductive network is more progressive, and the overall n value is relatively lower.

Overall, the key factors controlling variation in rock electrical parameters include preferential-channel development, fracture aperture, pore-throat scale differences, residual-water distribution locations, and the integrity of the continuous water-phase network. Among these factors, whether the continuous water phase connects the main fractures and key pore throats is the core factor determining abrupt resistivity changes and increases in n. Although irreducible water in vug corners, fine fractures, and poorly connected regions cannot fully maintain the overall conductive network, it delays rapid resistivity increases and affects staged variation in rock electrical parameters.

Therefore, rock electrical parameters in complex carbonate reservoirs are not fixed constants, but dynamically vary with displacement stage and fluid distribution state. Interpreting resistivity responses solely on the basis of water saturation can easily overlook the influence of pore structure and conductive-network connectivity. Combining microscopic displacement images with impedance measurement results can more accurately identify the pore-scale causes of non-Archie behavior and provide an experimental basis for water-invasion identification, remaining gas evaluation, and calibration of rock electrical parameters. The fitted saturation exponents should be regarded as effective parameters of the two-dimensional micromodels. Confinement of the current to a planar network may increase its sensitivity to the disconnection of key water-filled channels. The discussion therefore focuses on the relative differences and staged responses among the three pore structures rather than direct comparison with absolute values from three-dimensional rocks.

## 4. Conclusions

The experimental results show that pore structure controls dominant fluid-flow channels, residual-fluid distribution, and resistivity response during gas–water two-phase flow. In the fracture–vuggy type with large aperture, large-aperture fractures exert the strongest control on displacement. The flow is concentrated, and the resistivity response shows the greatest abruptness. The fracture–vuggy type with small aperture exhibits transitional characteristics under the combined effect of main-channel control and local trapping. In the vuggy type, fluid pathways are more dispersed. Bypass flow, side flow, and residual-fluid trapping are more pronounced. During gas–water displacement and water–gas displacement, resistivity shows staged variation. This variation is mainly controlled by the connection, disconnection, and restoration of the continuous water-phase conductive network. All three structures show non-Archie behavior during gas–water displacement. The stage-specific n values ranged from 2.62 to 13.10, 4.36 to 7.24, and 5.37 to 9.84 for the fracture–vuggy type with large aperture, fracture–vuggy type with small aperture, and vuggy type, respectively. Their overall n values were 7.26, 6.36, and 5.39. These results indicate that a more concentrated pore structure and a more prominent preferential conductive pathway lead to a greater deviation of the rock electrical relationship from the classical Archie model. The present method links pore-scale fluid distribution with the corresponding electrical response. Conventional rock electrical experiments provide bulk resistivity data but cannot directly show fluid migration. Visual micromodel experiments show fluid displacement but usually lack synchronous electrical measurements. Combining the two measurements helps explain how changes in fluid distribution and conductive pathways affect resistivity. This method provides an experimental basis for studying water invasion and identifying water-invaded zones in gas reservoirs. With suitable fluids and boundary conditions, it may also be extended to oil–water displacement and drilling-fluid invasion.

## Data Availability

The data presented in this study are available upon request from the corresponding author.
